# The relationship between lameness prevalence and pasture access in 659 dairy herds in Germany

**DOI:** 10.1371/journal.pone.0305536

**Published:** 2024-06-27

**Authors:** Anna Tillack, Roswitha Merle, Kerstin-Elisabeth Müller, Martina Hoedemaker, Katharina Charlotte Jensen, Alexander Bartel, Andreas W. Oehm, Marcus Klawitter, Annegret Stock

**Affiliations:** 1 Farm Animal Clinic, Division for Ruminants and Camelids, Unit for Internal Medicine and Surgery, School of Veterinary Medicine Freie Universität Berlin, Berlin, Germany; 2 Institute for Veterinary Epidemiology and Biostatistics, School of Veterinary Medicine Freie Universität Berlin, Berlin, Germany; 3 Clinic for Cattle, University of Veterinary Medicine, Foundation, Hannover, Germany; 4 Clinic for Ruminants with Ambulatory and Herd Health Services, Ludwig-Maximilians University Munich, Munich, Germany; 5 Zoetis Deutschland, Berlin, Germany; Michigan State University, UNITED STATES

## Abstract

Lameness in dairy cows is an expression of pain most likely originating from a claw disorder, causing impaired animal wellbeing and substantial economic losses for farmers. The aim of this study was to investigate the effects of access to pasture, time spent on pasture, and season on farm level lameness prevalence. The survey was part of a cross-sectional observational study, in which farms in three regions of Germany (North, East and South) were visited by study veterinarians. On each farm (total: 659, N: 240, E: 247, S: 172), management data were recorded, and cows were scored for locomotion, according to Sprecher. Median farm-level lameness prevalence (Score 3/5 or higher) was 29.4% (IQR: 18.7% - 42.0%), and 8.2% (IQR: 3.7% - 14.0%) for severe lameness (Score 4/5 or higher). Farm-level lameness prevalence continuously decreased with increasing time spent on pasture (up to approximately 10 hours per cow per day). On farms that did not offer their cows access to pasture lameness prevalence did not show a seasonal variation. On farms where cows had pasture access for up to three hours per day lameness prevalence peaked in autumn. In contrast, on farms offering their cows access to pasture beyond three hours per day the peak of lameness was observed in spring. Our results revealed that even short periods of pasture access of at least two hours per cow and day (on average per year) are beneficial for the locomotion of dairy cows.

## Introduction

Lameness represents one of the most important welfare problems of modern dairy farming and is the most common reason for early culling besides reproductive failure and mastitis [1, 2]. Lameness is a symptom characterized by gait abnormalities resulting from painful or functional disorders of the limb [3]. The nociceptive threshold in lame cows was shown to be considerably lower when compared with non-lame controls [4]. These observations underline that lame cows suffer from pain and demonstrate the negative impact of lameness on their physical and mental well-being. Next to welfare concerns lame cows cause substantial economic losses. These arise from supplementary costs for veterinary treatment, additional labour and losses due to premature culling [5], reproductive failure [6], and reduced milk yield [7] as well.

Studies from all over the world demonstrate varying prevalences of lameness. Recent numbers range from 3.8% in Australia [8], 15.7% in Germany [9], 28.3% in Canada [10] and 31.6% in the UK [11] depending on different features with respect to the geographical region and housing system. Access to pasture was shown to be beneficial for claw and leg health in dairy cows [12–14]. Lameness prevalence in countries with predominantly pasture-based dairy systems, e.g. New Zealand, is lower compared to countries with predominantly indoor housing [15]. Our previous analyses [16] showed that pasture access was associated with lower proportions of lame cows. The latter observation can be explained by the fact that pasturing offers more species-appropriate conditions for the bovine locomotor apparatus compared to systems in which cows are kept indoors the whole year round [13, 17]. The aspect that pasture access gives cows the possibility to exhibit normal behaviour meets consumer expectations with respect to the rising public demand for the well-being of food-producing animals. Although pasture-based dairy systems are implemented all over the world, the amount of access to pasture varies strongly, depending on soil quality and climatic conditions [18].

The aim of this study was to investigate the effects of access to pasture, time spent on pasture and season on farm level lameness prevalence.

## Materials and methods

This study was part of a larger cross-sectional observational study (PraeRi) [19] on housing conditions, health, and biosecurity on German dairy farms. The study design was described earlier by Jensen et al. [20] and Rittweg et al. [16]. No ethical approval and no animal experiment approval were necessary due to the legal regulations at that time in Germany. All participants gave their written consent to participate in the study and were informed that a) the data was analyzed anonymously and b) that they could quit the participation at any timepoint without any consequences.

### Farm recruitment and data collection

A total of 765 farms in three regions of Germany were enrolled in the PraeRi study: region North (N) with the federal states Schleswig-Holstein and Lower Saxony, region East (E) with the federal states Mecklenburg-West Pomerania, Brandenburg, Thuringia and Saxony-Anhalt and region South (S) with the federal state Bavaria. A sample of dairy farms was selected randomly by the state control associations of all regions; the exact sampling procedures are elaborated in Oehm et al. [21]. Within each region, all farms were grouped into three herd size categories, i.e., small, medium, and large depending on the number of milking cows. For each category within the study region a separate sample size was calculated as described by Rittweg et al. [16].

Farmers were contacted by postcard during the period from October 2016 until June 2019. In case of a positive response, they were contacted by phone and underwent a first survey in order to clarify if their farming system and management fulfilled the recruitment criteria: keeping dairy cows for commercial reasons (sale of milk). All farms fulfilling the criterion (N: 253, E: 252, S: 260) were visited by a team of trained veterinarians during the study period from December 2016 until August 2019. During the visit, data on animal level, compartment level, and farm level were collected by interviews, observations, and measurements and by backing up herd records for the twelve months before the visit. For all observations and measurements, standard operating procedures were used.

### Management based data

Herd size was obtained from the national traceability and information database for animals (HI-Tier). Housing type was recorded during the farm visit by the study team. Information on pasture access (number of months per year and hours per day with access to pasture for each of five lactation stages: early lactation, mid lactation, late lactation, early dry period, close-up period) were received in a face-to-face interview with the herd managers or farm owners.

### Animal based data

Information on breed, milk yield, and parity were collected from the national milk recording system (DHI) and from the data base HI-Tier. Lameness was assessed using a modified locomotion scoring system by Sprecher et al. [22] as presented in Table 1. Locomotion scores (LS) were dichotomised in two ways: “non-lame” (LS<3) and “lame” (LS≥3); “not or moderately lame” (LS<4) and “severely lame” (LS≥4). Observers followed a training in application of the scoring method and were evaluated once a year at inter-observer assessments to ensure a high level of agreement. Weighted kappa analysis of the observer trainings revealed that the interobserver agreement concerning the locomotion scoring varied between 0.39 and 0.63 [16]. However, these weighted kappa values were based on the 5-point scale and not on the dichotomized categories used here. Therefore, we assume a better agreement concerning these analyses.

**Table 1:** Modified visual locomotion scoring based on Sprecher et al. [22].

Score	Back Line		Gait/Posture
** **	**standing**	**walking**	** **
1	level-back	level back	normal
2	level-back	level-back	mildly affected gait
	level-back	arched-back	normal
3	level-back	arched back	clearly affected gait
	arched-back	arched-back	affected gait, short striding with one or more limbs
4	arched-back	arched-back	careful, favours one or more limbs
5	arched-back	arched-back	additionally: extreme reluctance or inability to bear weight on one or more limbs

### Statistical analyses

Statistical analysis was performed using R version 4.2.2 (R Foundation for Statistical Computing, Vienna). Results are reported with 95% confidence intervals. A significance threshold of 0.05 was used. Farm-level lameness prevalences are reported as median and interquartile range (IQR, i.e. 25% and 75% quantiles).

Herd size was categorized across regions in small (≤60 cows), medium (61–119 cows) and large (≥120 cows) farms. Farms were assigned to “free stalls with cubicle housing”, “straw-based free stalls”, and “housing systems with pasture access” if more than 80% of the cows were kept in the respective housing system on the day of the farm visit. If there was no such predominant housing system, the farm was assigned to the category “mixed”. The predominant breed on farm was classified accordingly (>80% of cows) as: “Holstein”, “Simmental”, and “Brown Swiss or others”.

The annual average time on pasture was calculated for all farms of the PraeRi study, indicating the average daily hours per year during which a cow had access to pasture on a farm. The average time spent on pasture was calculated as follows: for each of the five lactation groups the number of months was multiplied with 30 days and subsequently multiplied with the hours per day. The number for the five groups were averaged using a weighing factor for the length of the period (early lactation 100/397 days; mid lactation 100/397 days; late lactation 150/397 days; dry period 33/397 days; close-up 14/397 days) and divided by 365. In the following, two examples to illustrate the calculation: farmer A keeps his lactating cows in the barn and the dry cows on pasture night and day from beginning of May to end of October (6 months). Two weeks before the expected calving date, he brings the cows back to the barn. The annual average time on pasture for the cows of farmer A is 1.0 hours/day (= 24 * 6 * 30 * (33 / 397) / 365). Farmer B let her lactating cows on pasture after morning milking for 6 hours from May to September (5 months). The dry cows are on pasture night and day from May to October (6 months) and also are brought back in the barn two weeks before calving. The annual average time on pasture for the cows of farmer B is 3.2 hours/day (= [6 * 5 * 30 * (350 / 397) + 24 * 6 * 30 * (33 / 397)] / 365).

We grouped the herds into three categories: herds that had no access to pasture (0h), herds with up to three hours per day average (≤3h) and herds with more than three hours per day average (>3h).

Milk yield was expressed as energy corrected milk yield (ECM), calculated as follows: ECM = milk yield (kg) * [0.38* (fat%) + 0.21* (protein%) + 1.05] / 3.28 [23]. The mean ECM per cow of each day of testing was used to assess the average ECM per farm of all test days of the past twelve months before the farm visit.

The maps of housing system and pasture access for Germany were created using coordinates based on the postal codes of the visited farms. A 2-dimensional tensor spline was fitted with using a multinomial regression for housing system and a linear regression for the annual average time on pasture.

To examine how lameness prevalence in cows differed depending on their average yearly hours of pasture access, we fitted a restricted cubic spline. For visualizing the continuous seasonal effect on lameness prevalence, stratified by the categorical pasture access, we used cyclic cubic splines. All generalized additive models with splines were fitted using the R package mgcv (version 1.8–42).

## Results

### Study population and farm characteristics

In total, 659 farms were included in this analysis. Farm characteristics differed in the three regions as shown in Table 2. Overall, 94% of all farms had free stalls with cubicles or mixed housing systems. Most farms belonged to the category “large” (>121 cows), the predominant breed across regions was German Holstein, and average ECM for most farms was 25-30kg/cow (Table 2).

**Table 2:** Farm characteristics of participating farms and their distribution in region North, East and South.

	Level	Overall	North	East	South	Missing
**n**		659	240	247	172	
**Herd size (%)**	≤60	196 (29.7)	59 (24.6)	18 (7.3)	119 (69.2)	0.0%
	61–120	193 (29.3)	113 (47.1)	30 (12.1)	50 (29.1)	
	≥121	270 (41.0)	68 (28.3)	199 (80.6)	3 (1.7)	
**Housing system**^**1**^ **(%)**	free stall with cubicles	571 (86.6)	210 (87.5)	193 (78.1)	168 (97.7)	0.0%
	straw based	18 (2.7)	6 (2.5)	10 (4.0)	2 (1.2)	
	with access to pasture	15 (2.3)	9 (3.8)	6 (2.4)	0 (0.0)	
‍	mixed	55 (8.3)	15 (6.2)	38 (15.4)	2 (1.2)	0.0%
**Pasture access (%)**	0h	314 (47.6)	62 (25.8)	122 (49.4)	130 (75.6)	0.0%
	≤3h	157 (23.8)	61 (25.4)	78 (31.6)	18 (10.5)	
	>3h	188 (28.5)	117 (48.8)	47 (19.0)	24 (14.0)	
**Average ECM (%)**	<25kg/cow	154 (24.2)	43 (18.6)	39 (15.9)	72 (45.0)	3.5%
	25-30kg/cow	283 (44.5)	103 (44.6)	105 (42.9)	75 (46.9)	
	>30kg/cow	199 (31.3)	85 (36.8)	101 (41.2)	13 (8.1)	
**Breed**^**2**^ **(%)**	Holstein	441 (66.9)	222 (92.5)	214 (86.6)	5 (2.9)	0.0%
	Brown Swiss or others	87 (13.2)	17 (7.1)	31 (12.6)	39 (22.7)	
	Simmental	131 (19.9)	1 (0.4)	2 (0.8)	128 (74.4)	

n: Number of farms; ECM: energy corrected milk

^1^ ≥80% of the cows were kept in the respective housing system on the day of farm visit

^2^ ≥80% of the cows on the day of farm visit

### Lameness prevalence

Median farm level lameness prevalence (LS ≥3) was 29.4% (IQR: 18.7% - 42.0%). Prevalence for severe lameness (LS ≥4) was 8.2% (IQR: 3.7% - 14.0%).

### Access to pasture

**Fig 1:**
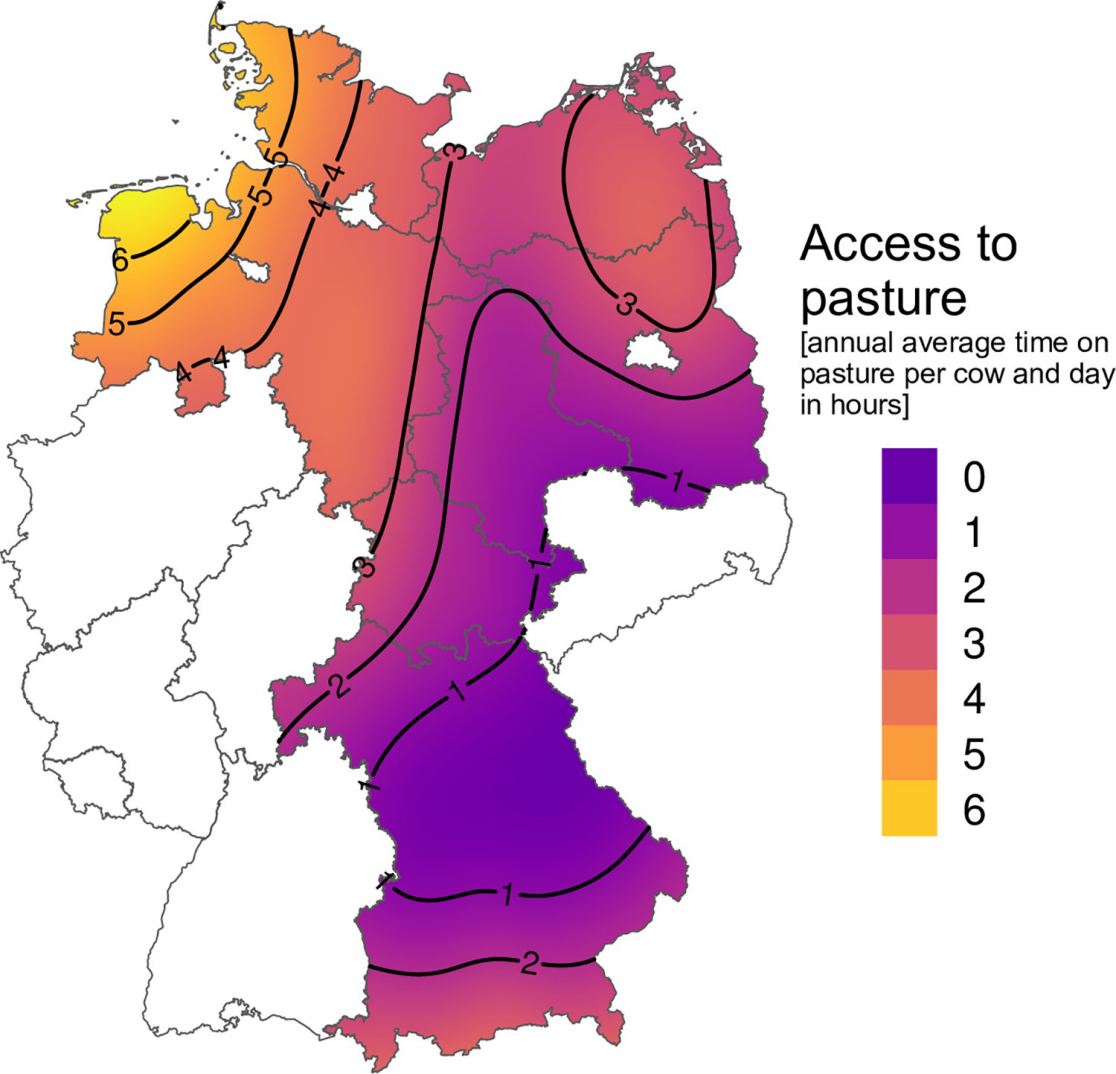
Annual average hours per day with access to pasture for dairy cows on 659 farms in Germany. The borders of the federal states are provided by GADM [24] for academic use under the GADM license.

Regarding all three regions, annual access to pasture differed (Fig 1). 314 (47.6%) farms did not provide any access to pasture (0h) for their cows. 157 (23.8%) farms offered an average time of up to three hours (≤3h) on pasture. Within this group, most farms (72% [113 of 157]) kept only their dry cows on pasture, whereas the lactating cows had no or limited pasture access. Dry cows were most often kept on pasture at night and day until they were brought back to the barn shortly before calving. All farms with an average daily pasture time of more than three hours (>3h; n = 188; 28.5%) kept lactating and dry cows on pasture. Within this group, the lactating cows had an average daily pasture time of ten hours for six months during summer. 58 farms kept their cows on pasture for more than 10 hours, so, during pasture season cows were only indoors for milking or if they decided to go indoors for feed or shadow.

### Time on pasture and lameness prevalence

**Fig 2:**
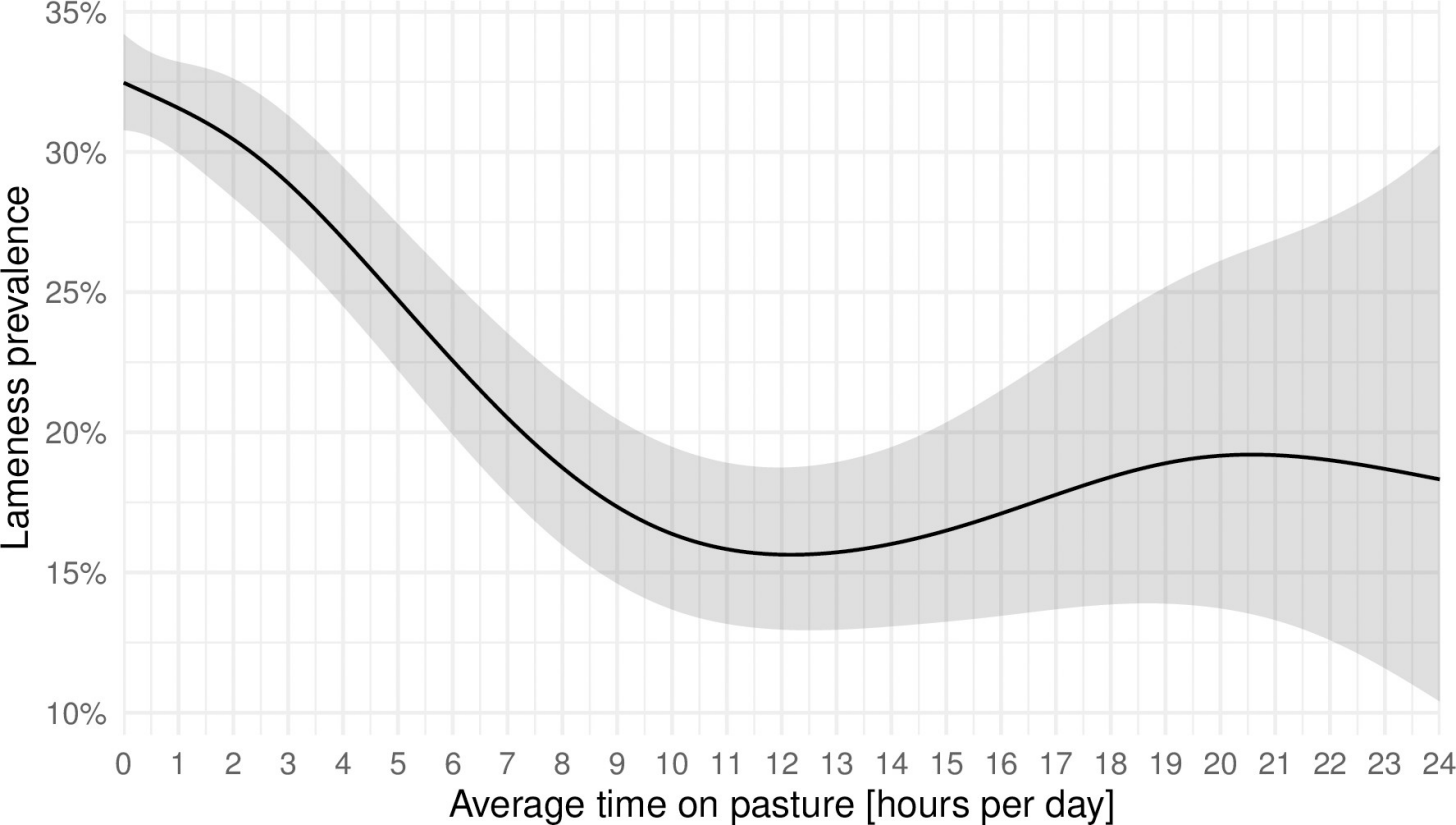
Effect of annual average time spent on pasture (hours per cow per day) on farm-level lameness prevalence. The annual average time on pasture indicates the average daily hours per year during which a cow had access to pasture on a farm. The effect of the annual average time on pasture on farm-level lameness was estimated using a restricted cubic spline. The shaded area represents the 95% confidence interval.

Lower lameness prevalence at farm level was observed with increasing time spent on pasture from 2 up to 10 hours per cow per day on average throughout the year. Beyond 10 hours per day and cow spend on pasture on an annual basis did not reveal any further decrease of farm level lameness prevalence (Fig 2). Further underlying data are provided in the supplements (S1 Table).

### Season and pasture

**Fig 3:**
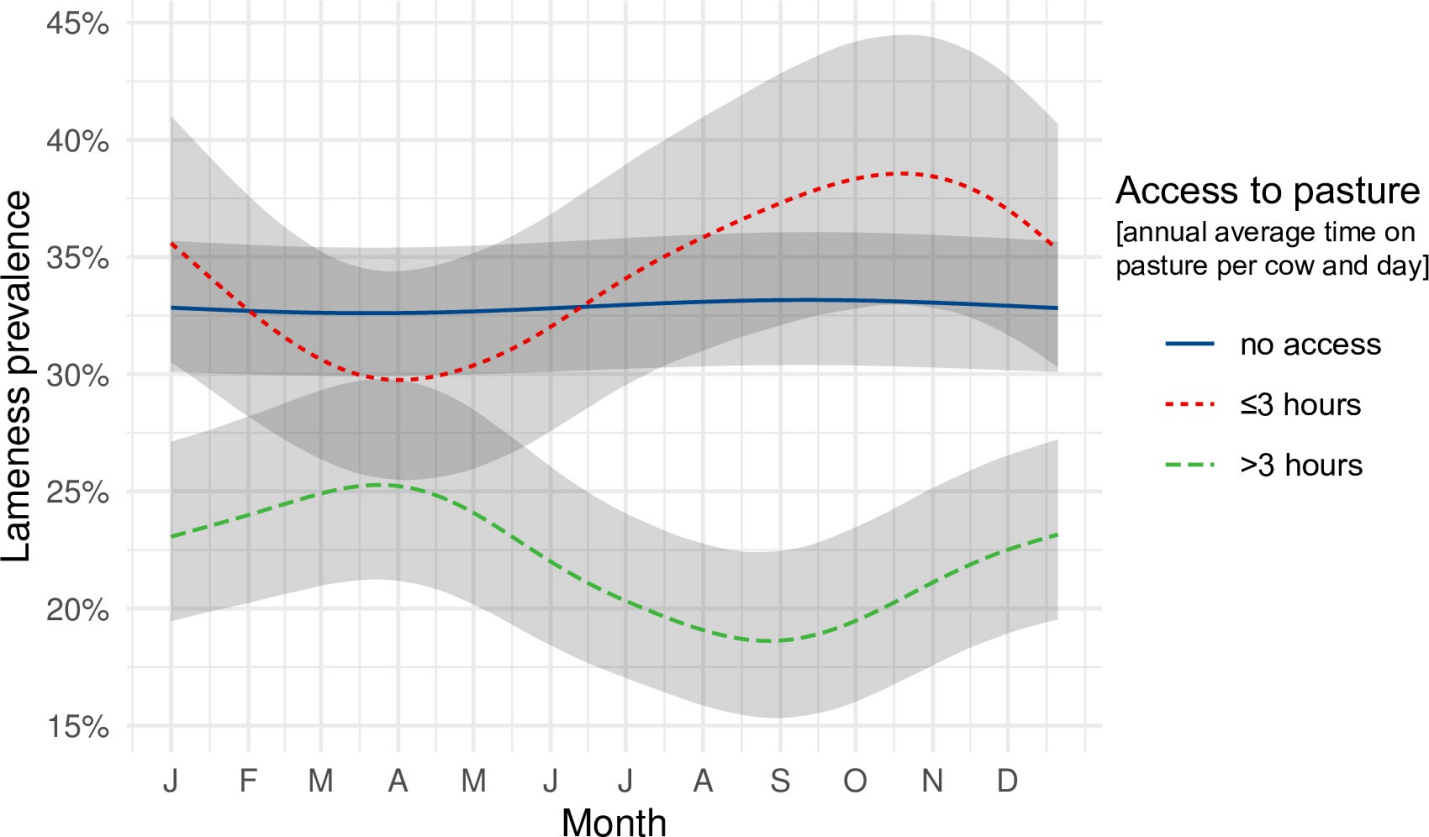
Effect of season on farm-level lameness prevalence stratified by annual average time spent on pasture per cow per day. The seasonal effect was modeled using a circular spline using the year day (1–365) of the farm visit. Farms were grouped into herds that had no access to pasture (0h), herds with up to three hours per day (≤3h) and herds with more than three hours per day (>3h). The shaded area represents the 95% confidence interval.

Lameness prevalence of the three groups (0h, ≤3h and >3h average daily pasture time) depending on the month of the year when lameness scoring was performed is displayed in Fig 3. The >3h-farms had the highest lameness prevalence rates in spring and summer and the lowest in autumn. The ≤3h-farms had an overall higher lameness prevalence, compared to farms with >3h access to pasture, with the lowest prevalence in spring and the highest in October. In 0h-farms no seasonal effect on lameness prevalence was observed.

## Discussion

### Lameness prevalence and study design

Lameness, with a prevalence of 29.4% is still a major problem on German dairy farms. Lameness prevalence as obtained within the PraeRi project was discussed by Jensen et al. [20] and is therefore only discussed briefly here. The lameness prevalence determined in this study was similar to the results of Dippel et al. [25]. Estimates of other studies conducted in Germany/Austria were lower [26, 27] or even higher [28]. Comparison of results from different studies should be handled with care, as various systems of mobility scoring were applied and farms participating in the studies differed substantially in housing conditions, management practices, and breeds. Compared to worldwide reported lameness prevalence on dairy farms, our results are within the upper range [10, 11, 29].

It must be noted that voluntary participation of farmers may have had an influence on the outcome (selection bias). However, the selection did probably not affect the effect of pasture on lameness which was examined here. The agreement of observers was fair to moderate which might have led to misclassification of cows since each observer only worked in one of the three regions (information bias). However, no significant differences in interobserver agreement were observed between the teams of the three regions. An information bias concerning the access to pasture is unlikely as information was retrieved directly from the farmers. Another possible information bias, which might have affected the estimated prevalence, is the fact that the scoring was not always performed on solid floors, but on slatted floors and straw as well. Scoring was rarely performed on pasture if not otherwise possible. This affected only a minor percentage of herds but might have led to an overestimation of the protective effect of pasture, since cows show better mobility on pasture, in our experience.

A strength of this study is the large number of participating farms across Germany, covering the different housing and management conditions typical for each region. Moreover, by performing the study in three different regions, an internal validation was carried out. The farms in the various regions of Germany included in the present study differed in management practices and farm structure e.g., herd size, main housing type, and pasture access practices. To this end, the three regions in our study represent different kinds of intensive dairy farming and provide a solid basis to extrapolate on the different farm structures. This is underlined by the wide range of our results for lameness prevalence, which also indicates that both, farms with low and high lameness prevalence rates are present in Germany [20].

### Time on pasture and lameness prevalence

In some countries e.g., Australia, year-round pasture access can be provided, whereas in northern regions e.g., Sweden, only two months per year are feasible [30]. In Germany regional and seasonal conditions determine pasture access practices. The distribution of the German agricultural area has to be considered when analysing the relationship between lameness prevalence and access to pasture. Region N was characterised by a balanced distribution of grassland and fields, whereas in the regions E and S fields are predominant. The latter fact resulted in a higher time on pasture for cows in region N compared to the other two regions (Fig 1 and Table 2). In Region S, tie-stall farms as well as organic farms are more common than in the other two study regions. Moreover, farms in Region S are the smallest study farms and often family run. However, in our study population (which included loose housing systems only) farms in Region S offer less time on pasture than the other two study regions: 75.6% of all farms in S had no access to pasture at all, whereas in N and E 25.8% and 49.4%, respectively, of farms had no access to pasture for dairy cows. Apparently, the formation of the landscape and the usability of the soil for agriculture have a bigger impact than farm characteristics. Perhaps small farms, that are usually situated in the middle of the village and not at the outskirts like bigger farms, have no pasture places nearby and workload would be too high to move dairy cows on a regularly basis between stall and pasture.

Our results indicate a decrease of lameness prevalence of 1.5–2% per hour for an average pasture time of 2 up to 10 hours per cow and day (Fig 2 and S1 Table). Hence, even limited access to pasture is beneficial regarding farm-level lameness prevalence. This is in accordance with the results of other studies [13, 14]. However, to our knowledge, this is the first study exploring the relationship between pasture time and lameness prevalence based on such a large sample size.

Additionally, farms offering their cows more than three hours pasture access showed a lower lameness prevalence, compared to farms with ≤3 hours pasture access and farms with all year indoor housing. The ground on pastures is softer and cleaner compared to artificial indoor surfaces with predominantly solid walking alleys. Thus, pasture provides a better environment for the digits (optimal pressure distribution, less slipping, better drying off), resulting in less claw horn lesions [17].

### Season and pasture

Next to all advantages, it must be noted that housing systems with access to pasture also entail some risks concerning claw and leg health. There are management and environmental risk factors to be mentioned, such as maintenance of cow tracks (e.g., stones) [31], and the strongly varying nutrient supply of pasture [32, 33]. Another important risk factor for lameness on pasture is season, since the development of many claw lesions has been associated with wet conditions [34].

Interestingly, the lameness prevalence showed different seasonal patterns in dependence of the average time that cows spent on pasture. On farms that did not provide any access to pasture at all lameness prevalence was almost constant all year long.

On farms with up to three hours (≤3) pasture access lameness prevalence was highest in autumn (after pasture season). In the existent literature is has been suggested that peaks of lameness prevalence in late summer/autumn are the consequence of heat stress in the month prior to the lameness event [35, 36]. Furthermore, incidence rates of many claw lesions (e.g. white line diseases, sole ulcers, infectious diseases, laminitis) are highest in summer [35]. Due to the time-delayed expression of the symptom lameness [35, 37], this becomes apparent later, in autumn.

Farms offering their cows more than three hours of pasture access (>3h) showed a completely different seasonal pattern with an increasing lameness prevalence in the housing period from September to April, which was also reported by others [38, 39]. The latter finding reflects once more the benefits of pasture access for cows in contrast to keeping the animals in confinement areas and might be explained by housing conditions that do not fulfil the requirements of dairy cows. In our experience, small farms with pasture-based dairy systems in summer do not invest as much money for husbandry and equipment compared to farms with indoor housing all year round. Bergsten et al. [30] concluded in their study that well-managed stall environment during the housing period has a greater impact on claw health than pasture.

## Conclusion

In conclusion, considering the fact that lameness in dairy cows is a reflection of painful conditions of the locomotor system, our results show that lameness prevalence in German dairy cows is unacceptably high, for welfare reasons. Providing pasture access for dairy cows, however, seems to exert beneficial effects on claw and leg health as reflected by lower lameness prevalence on farm level when compared to whole year indoor housing systems. In addition, lameness prevalence was even lower with increasing time spend on pasture, whereby even short periods of pasture access of at least two hours per cow and day (on average per year) are beneficial. Pasture-based systems demonstrated a clear seasonality with respect to lameness prevalence whereas systems of confinement housing displayed constant lameness prevalence during the whole year.

## Supporting information

S1 Table Lameness prevalence and farm distribution in the categories of the variable “annual average time on pasture per cow per day”. (XLSX)
